# Memristor-Based Neuromodulation Device for Real-Time Monitoring and Adaptive Control of Neuronal Populations

**DOI:** 10.1021/acsaelm.2c00198

**Published:** 2022-05-02

**Authors:** Catarina Dias, Domingos Castro, Miguel Aroso, João Ventura, Paulo Aguiar

**Affiliations:** † IFIMUP, Departamento de Física e Astronomia, Faculdade de Ciências, Universidade do Porto, Rua do Campo Alegre s/n, Porto 4169-007, Portugal; ‡ Neuroengineering and Computational Neuroscience Lab, INEB - Instituto de Engenharia Biomédica, Universidade do Porto, Rua Alfredo Allen, 208, Porto 4200-135, Portugal; § i3S—Instituto de Investigação e Inovação em Saúde, Universidade do Porto, Rua Alfredo Allen, 208, Porto 4200-135, Portugal

**Keywords:** hybrid bioelectronic systems, memristors, real-time control, neuroprosthesis, neuromodulation, implantable devices, in vitro neuronal populations

## Abstract

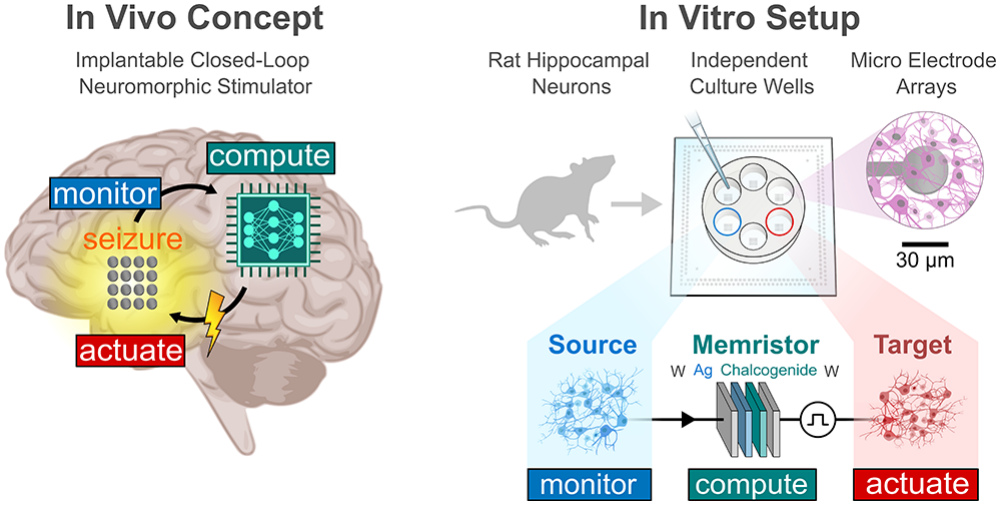

Neurons are specialized cells for information transmission and information processing. In fact, many neurologic disorders are directly linked not to cellular viability/homeostasis issues but rather to specific anomalies in electrical activity dynamics. Consequently, therapeutic strategies based on the direct modulation of neuronal electrical activity have been producing remarkable results, with successful examples ranging from cochlear implants to deep brain stimulation. Developments in these implantable devices are hindered, however, by important challenges such as power requirements, size factor, signal transduction, and adaptability/computational capabilities. Memristors, neuromorphic nanoscale electronic components able to emulate natural synapses, provide unique properties to address these constraints, and their use in neuroprosthetic devices is being actively explored. Here, we demonstrate, for the first time, the use of memristive devices in a clinically relevant setting where communication between two neuronal populations is conditioned to specific activity patterns in the source population. In our approach, the memristor device performs a pattern detection computation and acts as an artificial synapse capable of reversible short-term plasticity. Using in vitro hippocampal neuronal cultures, we show real-time adaptive control with a high degree of reproducibility using our monitor-compute-actuate paradigm. We envision very similar systems being used for the automatic detection and suppression of seizures in epileptic patients.

## 1 Introduction

Neurologic disorders are a major cause of death and disability worldwide, and their burden in society continues to increase with population aging and growth.^1^ Today’s therapeutic strategies still rely heavily on pharmacological approaches, with important problems regarding nonspecificity and side effects. Furthermore, progress has been notably slow in discovering new drugs for diseases such as Parkinson’s, Alzheimer’s, or epilepsy. Recognizing that neuronal function is intimately related to electrophysiology, attention is steadily growing toward a different therapeutic strategy: direct modulation of neuronal electrical activity. Deep brain stimulation for Parkinson’s disease, spinal cord stimulation for chronic pain, and cochlear implants for hearing loss are examples of success stories demonstrating the potential of this approach.

Neurotechnologies for therapies based on electrical modulation have, however, important challenges that still need to be addressed. Constraints in terms of size, power signature, signal transduction, and computational capabilities are currently limiting the progress in implantable medical devices. For example, the cost, risk (e.g., infection), and idiosyncrasy of surgeries solely for battery replacement cannot be underestimated. Development of novel devices should take into account that neurons and conventional electronics do not use the same electrical signals to encode information, and could take advantage of event-based operation modes to reduce energy consumption. Furthermore, any effective stimulation device requires adaptive computation capabilities to cope with the dynamic nature of neuronal activity. A device that is unable to dynamically respond to changes in neuronal activity patterns, falls short in its therapeutic potential and effectiveness.^2^

It is only natural to address these neuroprosthesis challenges by mimicking key properties of the nervous system’s solution to communication between neurons: the synapse. In this respect, memristive devices^3−10^ are a promising candidate to play the role of artificial synapses and integrate, as a core component, neuroprosthesis systems.^11−13^ Memristors are electrical components whose present conduction state depends on the electrical stimulus that has been previously applied to them. Although their properties rely on the particular combination of metal-insulator-metal materials used, the physical mechanisms behind conductance switching are typically related to the creation and rupture of nanoscale conductive filaments under the applied bias.^14−18^ Besides the low operation power involved in these switching mechanisms, the conductance state is also nonvolatile, staying unchanged when the power supply is removed, with additional benefits regarding power consumption. Among the most studied materials such as metal oxides (e.g., TiO_2_ and HfO_x_) and semiconductors (e.g., Si), the chalcogenide family (e.g., GeSe and Ag_2_S) has shown promising neuromorphic properties.^19−23^ Furthermore, as nanoscale two-terminal devices, their small feature size (<100 nm) enables high-density integration architectures and hardware implementation of powerful signal processors such as artificial neuronal networks.

**Figure 1:**
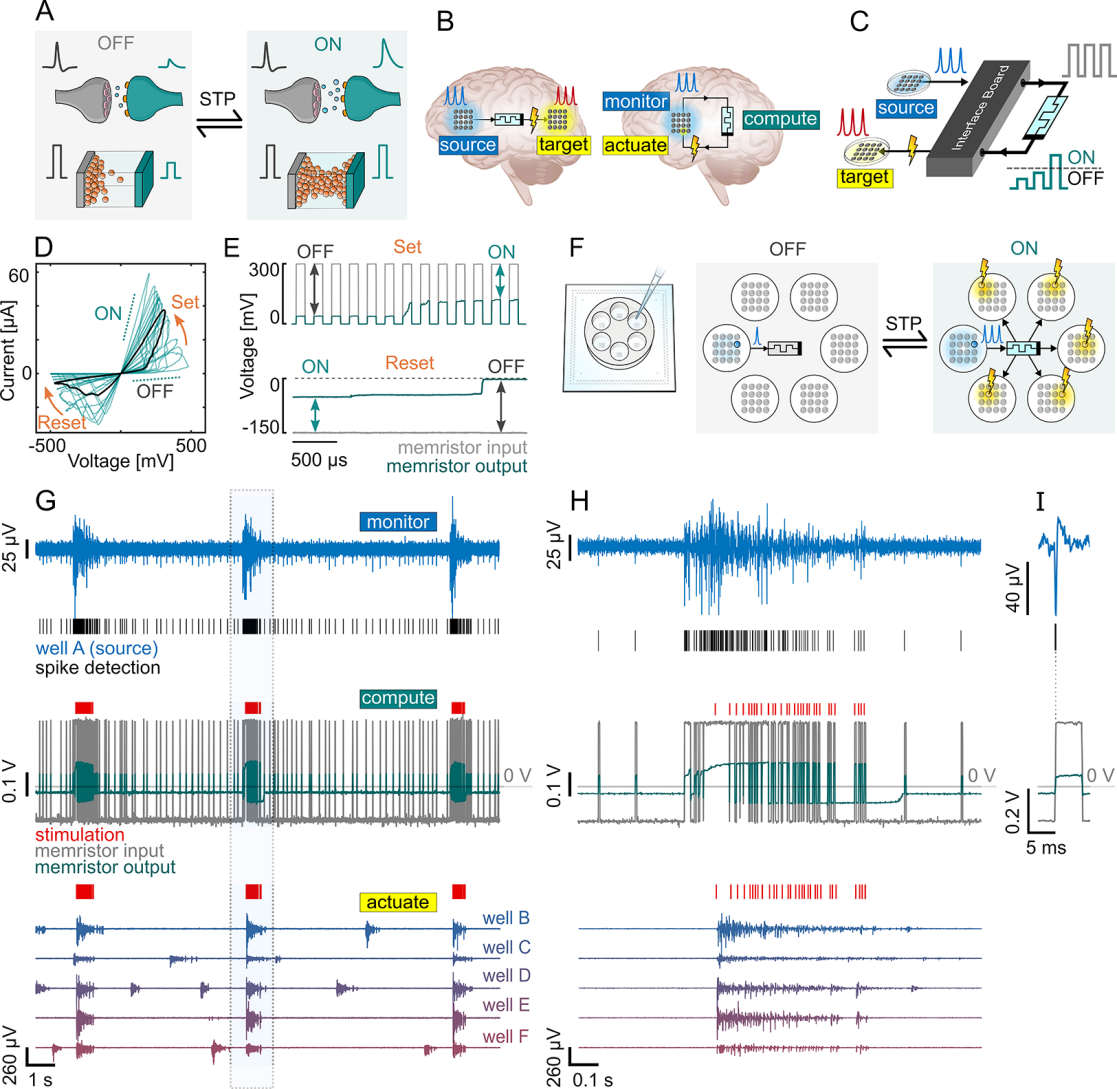
Real-time monitoring and adaptive control of neuronal populations using memristors. (A) Biological and memristive synapses share an analogous STP, with dynamics interchanging between low (OFF, left) and high (ON, right) conductance states as a function of the activity history. (B) Envisioned neuroprosthesis in vivo. The source and target neuronal population can be distinct (left) or the same (right), providing either way an effective monitor-compute-actuate paradigm through the memristor-based device. (C) In vitro implementation used in this work, with a hardware interface bridging neuronal and memristor signals. (D) Memristor hysteretic loop showing “set” under positive and “reset” under negative voltage []. The multiple cycles show the intrinsic variability of these types of devices, similar to the variability found in biological synapses. (E) STP dynamics with potentiation for repetitive positive pulsed (300 mV, 100 μs) stimuli (top) and recovery under constant negative (−150 mV) stimulus (bottom). (F) Schematic representation of the in vitro setting, integrating six physically independent neuronal cultures in a 6-well MEA (left) and a memristor to monitor the source population in real time and dynamically modulate the activity of five target populations (right). (G) Representative example of the real-time neuronal monitoring and modulation performed by the in vitro memristive interface. Actuation upon the target population is gated by sustained/consistent high-level activity at the source electrode (blue, top). The detected spikes (black traces, top) are converted to pulses and fed to the memristor (gray, middle). The memristor output (green, middle) increases when the source bursts, changing to the ON state. Consequently, this triggers electrical stimulation (red traces, middle and bottom) in the target population, which is therefore activated by the source. The recordings shown for the targets (bottom) are from electrodes neighboring the stimulation electrode. When the source bursts end, the memristor transitions back to the OFF state and stops propagating the source spikes to the target. (H) Zoom of panel G, evidencing the OFF–ON transition at the beginning of the source burst and the ON–OFF transition at the end. (I) Detail of a source neuronal spike and the associated detection performed by the interface board in real-time hardware (top). The spike creates a pulse that is applied to the memristor (bottom).

The dynamics of a memristor’s response to stimulation is analogous to that of synapses, presenting learned transitions from low to high conductivity states, and vice versa (Figure 1A). Importantly, the timescale of these transitions can be made analogous to the plasticity timescale of learning mechanisms in the brain, such as synaptic short-term plasticity (STP), which acts on the range from seconds to minutes. Furthermore, memristors can be integrated into the back-end-of-line of complementary metal-oxide semiconductor technologies in parallel with neuronal probe manufacturing.^24,25^ This means that memristive technology can leverage the recent developments in high-density microelectrode arrays (MEAs) and neuronal probes carrying hundreds to thousands of recording/stimulating electrodes at an unprecedented spatiotemporal resolution.^26^

The recognition of the exceptional combination of properties in memristors has fostered important proof-of-concept studies demonstrating the feasibility of direct neuron–memristor connection.^27−30^ Memristors can not only be made to respond to neuronal activity but they can also act as an effective interface between biological and artificial neurons, implemented either using software or using very large-scale integration hardware.^31^ A unidirectional, activity-dependent, direct coupling between two neurons in brain slices has also been recently achieved via organic memristive devices.^32^ In that study, however, the memristive devices only progressed from a low to high conductance state (linking the neurons), limiting the functional usefulness of the coupling system. Neuronal activity in these systems is recorded/stimulated using either MEAs,^27,28^ patch-clamp pipettes,^32^ or a combination of the two.^31^ Memristors not only emulate fundamental synaptic properties but they can also be combined to achieve nontrivial computations. Important examples, with particular interest for memristor-based neuroprosthesis, include the use of memristors to perform real-time processing of neuronal spikes^24^ or the use of memristor arrays to implement the filtering and identification of epilepsy-related neural signals.^33^ Although these proof-of-concept studies have covered important separate aspects of the potential role of memristors in implantable neuroprosthetic devices, to the best of our knowledge, no work has yet demonstrated the use of memristors in a fully functional configuration and in a clinically relevant setting.

It has already been well established that electrical stimulation can ameliorate the symptoms of epilepsy.^34^ As such, in this work, we take as our motivation the development of memristor-based closed-loop neurostimulators for intractable epilepsy. We believe such devices should perform three core tasks in a monitor-compute-actuate paradigm (Figure 1B): (1) online monitoring of a neuronal population prone to seizures, (2) real-time seizure detection, and (3) stimulation of specific (inhibitory or interfering) neuronal populations to suppress seizure progression. To produce our control setting, we developed a memristor-based interface (fully implemented in hardware) capable of performing real-time monitoring and adaptive coupling between two neuronal populations (Figure 1C). Our system establishes direct communication between neuronal populations (and not just individual neurons) and in vitro experiments are carried out with neurons from the hippocampus, a brain region frequently involved in epilepsy. The reversible short-term dynamics of the memristors is used both for the detection of network bursts (NBs) (a hallmark of epileptic seizures) and for the gating of electrical stimulation to the target neuronal population. NBs, defined as periods of strongly synchronized high-level activity in the neuronal population, share fundamental characteristics in both in vitro and in vivo conditions.^35^

## 2 Results and Discussion

In this work, we used commercial memristors composed of stacks of W/C + Ge_2_Se_2_/SnSe/Ge_2_Se_3_ Mix/Ag/Ge_2_Se_2_ Adhesion/W.^36−38^ These memristors rely on the movement of silver ions (Ag^+^) into channels within the active layer, which has been doped with carbon to enhance and optimize their properties, and are characterized by low-power binary switching. During the initial step of electroforming, under an applied positive voltage on the top electrode, Sn ions from the SnSe layer are generated and diffused into the active Ge_2_Se_3_ layer, where a metal-catalyzed reaction distorts the glass network to provide conductive channels for the movement of Ag^+^. Because the amount of Ag within the channel determines the resistance of the device, the resistance is then tunable by the movement of silver into or away from these channels by applying positive (“set”, ON state) or negative (“reset”, OFF state) potential, respectively (Figure 1D).^37^ The typical device-to-device variability is reflected in the electrical *I*–*V* behavior, where a larger hysteresis translates into a higher separation between the resistance states (Figure S1). Notably, these types of memristors are capable of short-term memory dynamics. Of particular importance for this work is the reversibility of the ON–OFF conductance transitions. With conditioning positive pulses and constant negative voltage, we were able to produce interchangeable “set” and “reset” transitions, respectively, with adequate timescales for a neurobiology setting (Figure 1E). These transitions are shown in the cumulative voltage increase from low (OFF state) to high (ON state) at each positive pulse (“set”) and the change from a constant negative voltage (ON state) to almost zero (OFF state) under constant applied bias (“reset”). This cumulative voltage increase after each positive pulse produces functionally relevant STP behavior (Figure S2). Aiming at population-level control, our designed system relies on MEAs, which allow long-lasting recordings and modulation of neuronal activity (as opposed to patch-clamp electrodes). As to discriminate the memristive device’s specific contribution to neuronal activity modulation, we used neuronal cultures on MEAs with a 6-well configuration, forcing the existence of six physically independent neuronal populations (Figure 1F). Communication between the source population/well and the remaining populations/wells was mediated by the memristor, whose selectivity commanded electrical stimulation of the target populations according to the patterns of activity detected in the source population.

Currently, there are still no memristors available working in a voltage/current range compatible with a direct connection to neurons (μV and nA). As such, and for now, instead of a direct/passive circuit neurons-microelectrode-memristor-microelectrode-neurons, our system includes hardware (e.g., amplifiers and electrical stimulators) to translate between microelectrodes and memristors’ voltage amplitudes (Figure S3).

### 2.1 Memristive Interface Detects and Selectively Responds to Network Bursting Activity in Real Time

Initially, the memristor is in a low conductance state (OFF), analogous to a weak synaptic connection. Bursting activity at the source electrode induced a gradual increase in the memristor conductivity, changing its state to ON. When the memristor was ON, source spikes triggered electrical stimulation in the target population, inducing them to fire with the source population (Figure 1G,H). Importantly, the established neuronal coupling/modulation is reversible and the connection is dissolved when the spiking rate of the source decreases back to baseline activity. Note that the evolution of the memristor’s state is automatic and unsupervised, which means that no additional system is used to actively change its conductance—the memristive interface does the NB detection computation autonomously. The time needed for the transition (<100 ms) is still low enough for the burst triggered in the targets to be in synchrony with the source burst, as the latter can last between several hundreds of milliseconds to seconds.

The memristor receives a pulse for every spike in the source electrode (Figure 1I). A conditioning circuit (Experimental Section and Figure S3) was fine-tuned prior to the experiments to guarantee that the different patterns of activity exhibited by the neurons (patterns of received pulses) induce the memristor to interchange between its conductive states, ensuring the desired selective response to bursts. We lowered the amplitude of the arriving signal to assure that each pulse had a positive amplitude, lower than the memristor’s “set” threshold. A pulse with an amplitude above the “set” threshold would immediately transition the memristor state to ON with a single source spike, eliminating any computation capabilities. When no spikes were being sent, the voltage across the memristor was negative with a small amplitude, slowly changing its resistance back to OFF. These parameters—amplitude of the pulse and negative baseline level—were optimized a priori and kept unchanged for all the experiments. This was performed taking into consideration the natural stochasticity of the memristive behavior. In terms of material dynamics, the positive pulses are gradually and cumulatively creating a metallic filament across the device, whereas the negative constant voltage is slowly dissolving the filament. Note that the latter would not be needed in the case of volatile memristors.

### 2.2 Memristive Interface Promotes Robust Coupling and Modulation of Neuronal Populations

**Figure 2:**
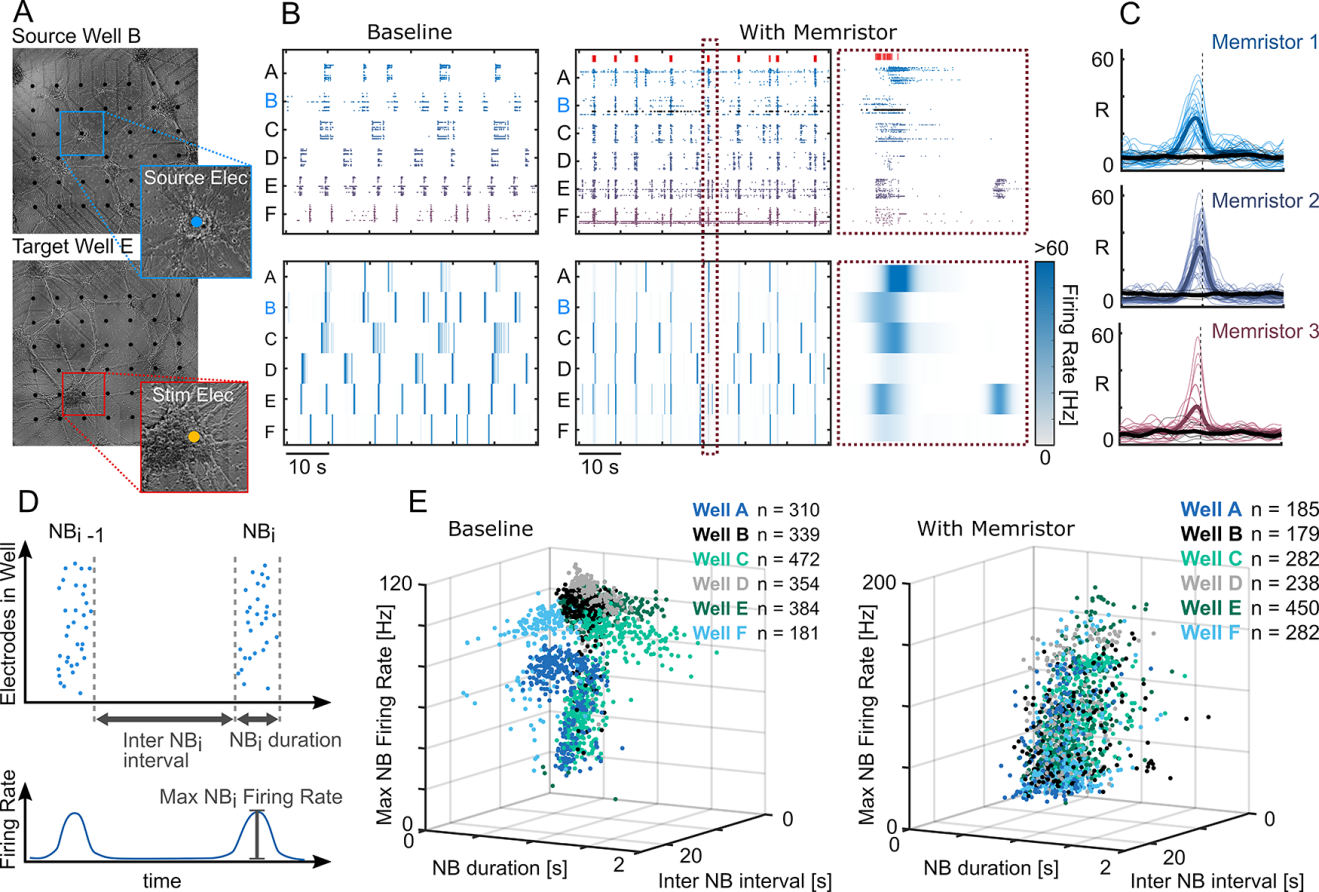
Real-time coupling and modulation of neuronal networks using a memristor. (A) Representative source and target neuronal networks cultured in different wells (scale bar = 500 μm). (B) Raster plot (top) and average firing rate per electrode on the distinct wells (bottom); the left and right panels correspond, respectively, to the system without and with the memristor-mediated connection. High-frequency spikes in the source electrode (black dots, top right) correlate with NBs in the source well (well B, in this representative example). (C) Synchronization of high-frequency activity between the source and targets is evidenced by the CC curves between the average firing rate per electrode on the wells (B, bottom). There is a CC curve for each source–target pair per trial for the three memristors tested (Memristor #1: 6 trials, 3.7 ± 1.7 min each; Memristor #2: 8 trials, 6.8 ± 4.2 min each; and Memristor #3: 6 trials, 11.9 ± 4.5 min each). The colored lines represent the CC curves for the trials with the memristor, gray curves are for trials without the memristor. The thicker lines represent the average. (D) Diagram describing the three parameters used for the network activity fingerprints: NB duration, interval, and maximum firing rate. (E) Memristive interface modulates the dynamics of the network activity in the target wells. Each dot represents an NB in a given well. Before inserting the memristor (left), the NBs of each well had consistent separable features (duration, interval, and maximum firing rate). With the memristive interface, the activity fingerprints of the source (well B) and target wells are indistinguishable (right), showing that the network dynamics that govern the target populations were modulated by the source.

The memristor communicates with the neuronal cultures through a source electrode in the source well and a stimulation electrode in each target well (Figure 2A). The bursting periods detected by the memristor are associated with network-wide events that dominate the culture activity (Figure 2B), referred to as NBs. Although the memristor responds to the spikes from a single source electrode because this extracellular electrode records from multiple neighboring cells^39^ (see zoom in Figure 2A), the memristor is in fact tuned to detect the patterns of strong and synchronous network bursting activity. When there is no memristor-modulated communication between the source and target wells, the network activities of the six cultures are independent of each other (Figure 2B, left). When the memristor is inserted, the NBs of the source are detected due to the sudden increase in activity in the source electrode. This activity pattern sets the memristor state to ON, closing the modulatory bridge between the source and target wells via electrical stimulation. In turn, the electrical stimuli applied to the stimulation electrodes modulate neuronal activity in the target wells, thus establishing a communication pathway between previously isolated neuronal populations (Figure 2B, right). The short-term memory of the memristor operates at the temporal scales of the NB dynamics and guarantees that after the NB finishes in the source well, the memristor transitions back to OFF, avoiding isolated source spikes to interfere with the activity of the target populations.

Three different memristors were tested in separate trials, each monitoring a different source well. Qualitatively, the three memristors maintained a strong coupling between the high-frequency activity patterns in the source and target wells when compared to the baseline (without memristor). To quantify this dynamic coupling, we computed the cross correlation (CC) between the mean firing rate of each source–target well pair for each trial (Figure 2C). Despite the inherent variability of the memristor, the CC curves showed a significant correlation between the firing rates of source and target wells, with a delay in the millisecond range. The memristive interface was thus capable of maintaining a robust low-latency coupling of specific activity patterns between independent neuronal populations.

Besides quantifying the temporal coupling of the different networks, we also evaluated the impact of the memristive control on the modulation of the activity fingerprints of the target networks. Specifically, we focused on the changes in properties such as duration and intervals between NBs, and maximum firing rates inside each NB (Figure 2D). In the system without the memristor, each well had its own, independent, network activity fingerprint. In the memristor-based system, the network activity profile in all the target wells followed, within physiological bounds, the command activity of the source well (Figure 2E). This shows that the dynamic modulation provided by the memristor aligned the activations of the target populations with the NBs of the source population. In the context of the detection-suppression system for epilepsy, the target neuronal population (or brain region) would be chosen so as to have an inhibitory (or interfering) effect on the source population.

## 3 Conclusions

Neuromorphic devices provide promising properties to address the size, power, and computational requirements critical for innovative implantable neuroprosthesis. Memristors, in particular, exhibit neuronal-like dynamics and can be tuned to operate on equivalent temporal scales. Here, we used memristors as an effective alternative to microprocessors to monitor biological neuronal populations and selectively modulate their activity in a long-term autonomous setting. Our in vitro memristor-based system performs the three core tasks required for an efficient feedback neurostimulator—monitor, compute, and actuate—and it does so with a nanosized, low-power, neuromorphic computing element. The robust low-latency detection and modulation of network activity patterns presented in this proof-of-concept is fundamentally important for an implantable neuronal stimulator in many clinically relevant contexts. Here, the computations were performed by a single memristor, allowing the detection of simple neuronal patterns. Future studies should focus on the integration of memristive arrays with biological neurons to perform real-time detection of more refined patterns of activity. Also, here the communication between neurons and memristors was mediated by an interface board and a conditioning circuit. With memristors’ diversity increasing every day, future improvements will focus on establishing a passive direct connection between neurons and memristors.

## 4 Experimental Section

### 4.1 Memristive Devices

The memristive devices used were acquired from KNOWM Inc., and are composed of stacks of W/C + Ge_2_Se_2_/SnSe/Ge_2_Se_3_ Mix/Ag/Ge_2_Se_2_ Adhesion/W.^36−38^ They are analog devices with very low switching energy and fast switching response. The inherent threshold voltage of these devices varies in the range of approximately 0.25–0.45 V (Figure 1C). As the plasticity dynamics can be tuned by the applied frequency, amplitude and duration of pulse stimulation, to properly choose the values of the components in the electrical circuit (Figure S3), we performed a preceding characterization of the devices’ response to pulse stimulation, as can be seen in Figure 1D.

### 4.2 Cell Culture

All the experiments were performed in accordance with the European legislation for the use of animals for scientific purposes and protocols approved by the ethical committee of i3S. The Animal Facility of i3S is licensed by the Portuguese official veterinary department (DGAV, Ref 004461), complies with the European Guidelines (Directive 2010/63/EU) transposed to Portuguese legislation by Decreto-Lei no 113/2013, and follows the FELASA guidelines and recommendations concerning laboratory animal welfare. Embryonic (E18) rat hippocampal neurons were seeded and cultured at a density of 5 × 10^5^ cells/well on a 6-well round chamber MEA with a macrolon ring 10 mm high (256-6well MEA200/30iR-ITO-rcr) (Multichannel System MCS, Germany). The 6-well MEA electrode has an array of 7 × 6 TiN electrodes in each well, with a total of 252 recording electrodes. Half-medium changes of Neurobasal TM Plus (Thermo Fisher Scientific) were performed every 2–3 days.

### 4.3 Electrophysiological Recordings

The experiments were performed at 11 and 13 DIV using the MEA2100-256 system (Multichannel System MCS, Germany) at a sampling rate of 10 kHz. The electrophysiological signals were high-pass filtered at 200 Hz. The recordings were acquired using Experimenter software from MCS. Cell culture conditions (37 °C and 5% CO_2_) were maintained with a stage-top incubator (ibidi GmbH, Germany) adapted to the headstage of the MEA2100-256 system.

### 4.4 Real-Time Spike Detection

The detection of spikes in the source well was performed in real time (latency of less than 20 μs) with the digital signal processor (DSP) included in the interface board of the MEA2100-256 system, using a threshold crossing method on the already filtered signal. The negative threshold had five standard deviations (sometimes manually tuned at the beginning of the trials). Such a threshold is significantly higher than the background noise, ensuring that only neuronal spikes are sent to the memristor. The DSP was configured using Experimenter software before starting the trials, making it independent of the computer from there on. The interface board sends a Transistor-Transistor Logic (TTL) pulse to the memristor whenever the potential at the recording electrodes exceeds a pre-established threshold. The duration of the TTL was adjusted at the beginning of the trial to either 1, 5, 10, or 20 ms. Once configured, the entire system was fully independent of the computer.

### 4.5 Mediating Electrical Circuit

The 3.3 V TTL pulses arriving from the interface board (digital output) were attenuated to fit the memristor operation range (millivolt range) using a dedicated circuit (see Figure S3 in Supporting Information). The circuit was tuned to ensure that only a high frequency of incoming pulses would set the memristor from OFF to ON in the required temporal scales. Also, the baseline level was set to a small negative value that ensured the transition from ON to OFF after a significant period without neuronal spikes. The signal processed by the memristors was converted back to the operating range of the digital input of the interface board.

### 4.6 Electrical Stimulation

A stimulation electrode was selected for each target well. The electrical stimulus was a negative monophasic voltage pulse of 500 mV and 200 μs. The electrical stimulation was triggered for all the stimulation electrodes every time the interface board received a high amplitude TTL pulse (above 2 V) coming from the memristor. This way, when the memristor was ON, each spike detected in the source well triggered an electrical stimulation in each target well. The interface board operates at 50 kHz, meaning that it takes 20 μs to activate the stimulation once the memristor response is received. The closed-loop latency associated with the spike detection performed by the DSP and the activation of the stimulator is lower than the sampling period (0.1 ms). Stimulation artifacts are removed by blanking the recording electrodes for a few instants (2 ms) after each stimulus (blanking operation available in the MEA2100-256 system).

### 4.7 Experimental Protocol

The experiments began with a baseline recording of 10–30 min without memristor intervention. An electrode that had both bursting activity and isolated spikes was chosen as the source electrode and its well, therefore, as the source well. To select effective stimulation electrodes, the four most active electrodes of each well were individually stimulated with 20 electrical pulses at an interval of 10 s. For each well, we chose the electrode that triggered a response in the largest number of neighboring electrodes. The memristor was then inserted into the conditioning circuit connected to the interface board. Before starting the memristor experiment, the duration of the TTL pulses was adjusted to either 1, 5, 10, or 20 ms to assure proper STP on the desired temporal scales. Each trial had a maximum duration of 15 min, but could be stopped earlier if the memristor got stuck in an ON or OFF state for several minutes. The entirety of the recordings was considered in the analysis, including the periods where the memristor was apparently stuck in a state. A computer running MCS Experimenter software (Multichannel System MCS, Germany) was used solely to (1) record neuronal data and the stimulation time stamps for later offline analysis and (2) upload to the MEA2100 system the parametrization for the stimulator and the DSP (after this upload, the closed-loop system itself is independent of the computer).

### 4.8 Signal Processing

The neuronal signals were filtered with a 200 Hz high-pass and spike detection was performed for all the electrodes (not in real time) using positive and negative six standard deviation thresholds with 3 ms dead time. The mean firing rate of each well was obtained by convolution of the spike trains of the electrodes with a Gaussian kernel of 0.05 s sigma and averaging across electrodes. The cross-CC curves were calculated between the mean firing rate profiles of each source–target well pair. The NBs consisted of periods when several electrodes burst simultaneously. Events that included less than five bursting electrodes were not considered. To identify the bursting periods of each individual electrode associated with the NB, we considered groups of at least five spikes with an interspike interval of less than 100 ms. The interspike interval considered for the burst detection in individual electrodes is larger than what is typically considered for bursting neurons (usually around 5 ms) because we wanted each electrode burst to encompass the full contribution of that electrode to the NB (instead of multiple bursts in the same electrode for the same NB).

## Supporting Information Available

*I*–*V* hysteretic behavior of discrete memristive devices; memristive STP behavior; and schematic of the conditioning electrical circuit (PDF)

## Notes

The data that support the findings of this study are available from the corresponding author upon reasonable request.
