# Supplementary material for: Memristor-Based Neuromodulation Device for Real-Time Monitoring and Adaptive Control of Neuronal Populations
Source: ACS Appl Electron Mater. 2022 May 2;4(5):2380–7. doi: 10.1021/acsaelm.2c00198 (PMC9778128; doi:10.1021/acsaelm.2c00198)
Supplement: Supplementary file 1 — el2c00198_si_001.pdf [file el2c00198_si_001.pdf]

Supporting Information

A memristor-based neuromodulation device for real-time monitoring and adaptive control of neuronal populations

Catarina Dias[1,§], Domingos Castro[2,3,§], Miguel Aroso[2,3], João Ventura[1], and Paulo Aguiar[2,3,]*

[1] IFIMUP, Departamento de Física e Astronomia, Faculdade de Ciências, Universidade do Porto, Rua do Campo Alegre s/n, 4169-007 Porto, Portugal

[2] Neuroengineering and Computational Neuroscience Lab, INEB - Instituto de Engenharia Biomédica, Universidade do Porto, Rua Alfredo Allen, 208,

4200-135 Porto, Portugal

[3] i3S - Instituto de Investigação e Inovação em Saúde, Universidade do Porto, Rua Alfredo Allen, 208, 4200-135 Porto, Portugal

§ These authors contributed equally to this work and are listed in alphabetical order

* Corresponding author

E-mail: pauloaguiar@i3s.up.pt

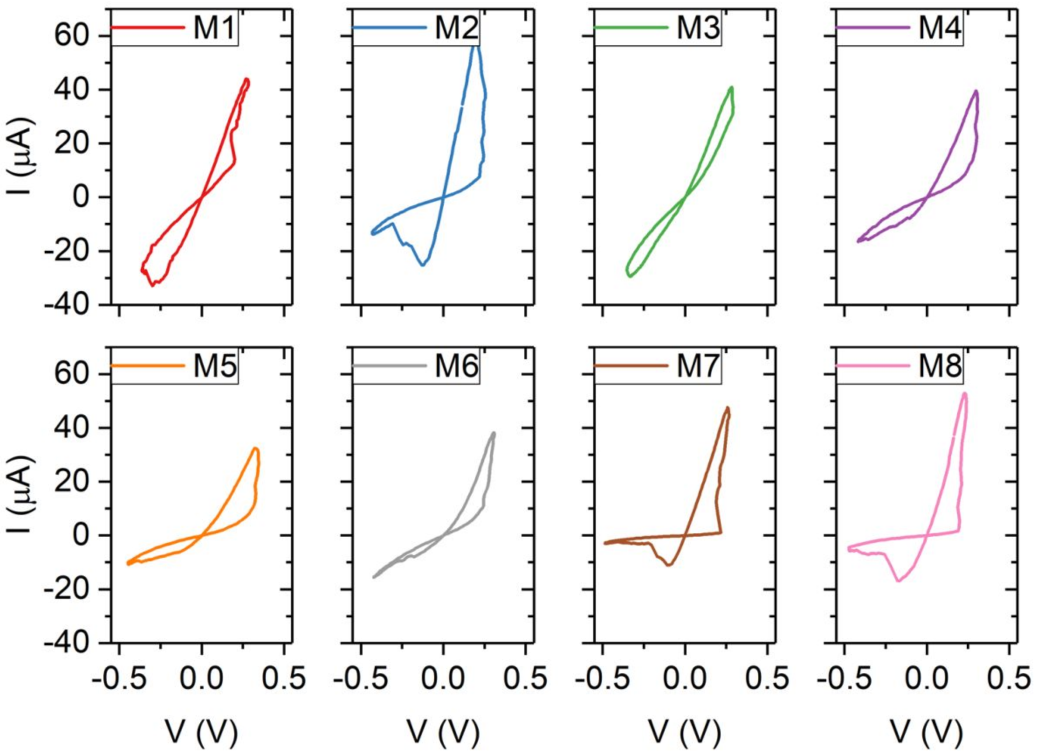

Figure S1. Representative electrical I-V hysteretic behaviour of 8 discrete memristive devices in the same chip. The devices show low operation power and the typical device-to-device variability. A larger hysteresis translates in a higher separation between the resistance states.

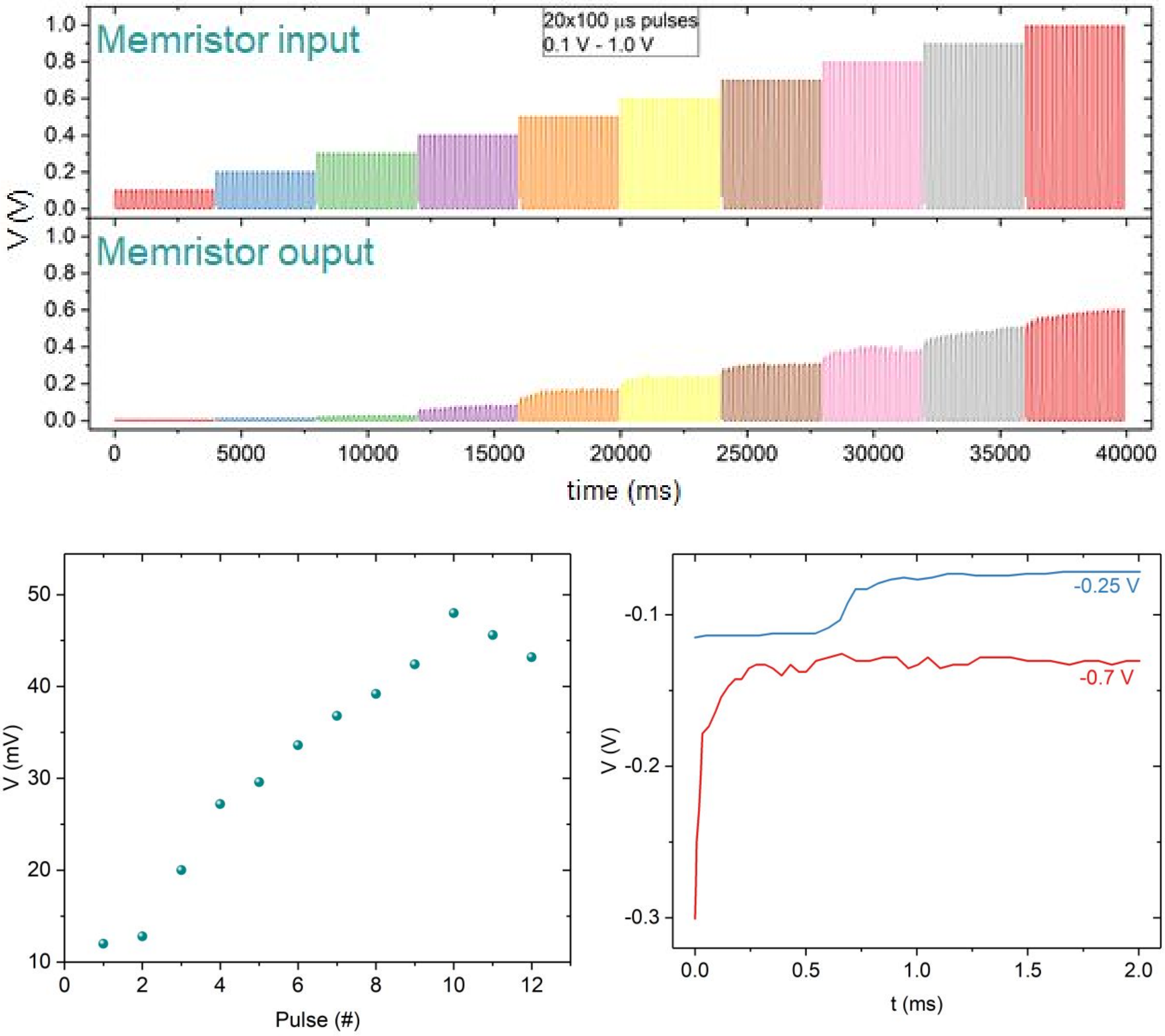

Figure S2. Representative short-term plasticity (STP) behaviour. Effect of the voltage amplitude (0.1 – 1.0 V) on the resistance change at fixed pulse number (20), duration (100 µs) and frequency (top). Cumulative memristor voltage increase (resistance decrease) after each 320 ms pulse at 2 Hz (bottom left). Effect of the negative voltage amplitude (-0.25 and -0.7 V) on the resistance change time (bottom right).

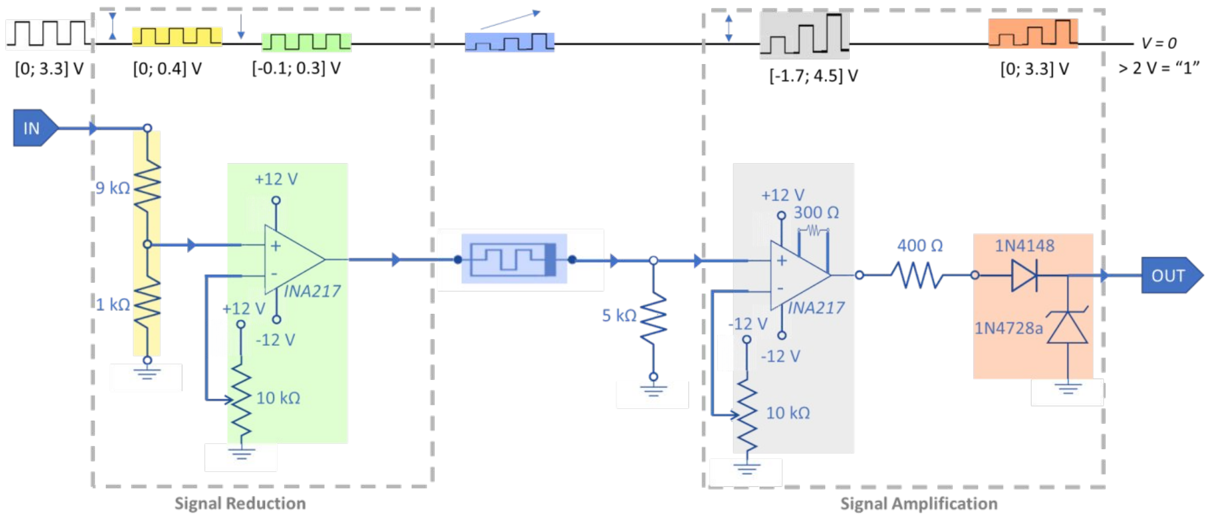

Figure S3. Electrical circuit used to connect the memristor with the interface board of the MEA2100 System from MultiChannel Systems (bottom) and a schematic representation of the TTL signal across the circuit (top). The first block is responsible for the transformation of TTL signal to the lower operation voltage range of the memristor. A voltage divider and a low-noise instrumentation amplifier shift the pulse to have both a positive (for "set") and a negative (for "reset") component. The last block amplifies the signal at the memristor output, for it to be read by the interface board as a logical "0" when the memristor is OFF (TTL < 2 V) and a logical "1" when the memristor is ON (TTL > 2 V). The diodes are only present as a precaution, for protection of the data acquisition system.
